# Time-Frequency Energy Sensing of Communication Signals and Its Application in Co-Channel Interference Suppression

**DOI:** 10.3390/s18072378

**Published:** 2018-07-21

**Authors:** Yue Li, Liang Ye, Xuejun Sha

**Affiliations:** 1 School of Electrical Engineering, Heilongjiang University, Harbin 150080, China; 2017021@hlju.edu.cn; 2 School of Electronics and Information Engineering, Harbin Institute of Technology, Harbin 150080, China; coldwound@163.com

**Keywords:** time-frequency energy sensing, STFT, Choi-Williams distribution, time-varying filter, co-channel interference

## Abstract

As the number of mobile users and video traffics grow explosively, the data rate demands increase tremendously. To improve the spectral efficiency, the spectrum are reused inter cell or intra cell, such as the ultra dense network with multi-cell or the cellular network with Device-to-Device communications, where the co-channel interferences are brought and needs to be suppressed. According to the time-frequency energy sensing to the communication signals, the desired signal and the interference signal have different energy concentration areas on the time frequency plane, which provide opportunities to suppress the co-channel interference with time varying filter. This paper analyzes the time-frequency distributions of the Gaussian pulse shaping signals, discusses the effect of the analyzing window length on the time-frequency resolution, exploits the equivalence between the time frequency analysis at the baseband and at the radio front end, and finally reveals the advantages of the proposed masking threshold constrained time varying filter based co-channel interference mitigation method. The pass region for the linear time varying filter is generated according to the time-varying energy characteristics of the I/Q separated 4-QAM pulse shaping signals, where the optimum masking threshold is obtained by the optimum-BER criterion. The proposed co-channel interference suppression method is evaluated in aspect of BER performance, and simulation results show that the proposed method outperforms existing methods with low-pass or band-pass filters.

## 1. Introduction

With the development of mobile internet, the wireless data transmission rate demands increase rapidly [1]. The main bottleneck to achieve such a requirement is the shortage of available licensed spectrum. To solve this problem, one effective solution is to reuse the same resources in heterogeneous networks, which is named non-orthogonal resource sharing. For example, the ultra density network is an effective way to improve the spectral efficiency. Since the small cell base stations generally reuse the same channels with the main base station, strong co-channel interference overlapping on the time-frequency plane may be brought to the ultra density network [2,3]. In addition, in Device-to-Device communications (D2D) underlaying cellular network, the signals of cellular users and D2D users with the non-orthogonal resource sharing mode are also superposed in time-frequency domain [4]. In both cases, one important issue is to suppress the non-orthogonal or co-channel interferences while maintaining the desired signal’s frequency characteristic.

In current communication systems, the signals are analyzed by Fourier transform, which shows what frequency components one signal contains, but does not tell when the frequency components exist. In contrast to Fourier transform, time frequency analysis is a powerful tool which shows the energy concentration characteristics of non-stationary signals on the time-frequency plane. The time-frequency distributions have been utilized in radar signal analysis, the linear frequency modulated signal detection, and the PSK/FSK signal distribution by extracting the signal features such as instant frequency, instant energy and instant phase [5,6,7,8,9]. Nicholas et al. [5] applied the proposed time-frequency distribution based on compressed sensing to radar backscattered signals for signature analysis, where the compressed sensing joint time frequency distribution works well for signals since the cross-term energy and signal energy are well separated in the ambiguity plane. Other authors [6,7,8] used the time frequency distribution to solve the interference problems in GNSS systems, and real GNSS data collected in the presence of jamming were used to demonstrate the effectiveness of time frequency-based antijam approaches. Han et al. [9] used Wigner-Ville distribution slice and fractional Fourier transform to detect the moving target in SAR system, and Samir et al. [10] addressed the problem of direction of arrival estimation and blind source separation for nonstationary signals in the underdetermined case, and used high-resolution quadratic time-frequency distributions to deal with the problem. Zhang et al. [11] used Choi-Williams time-frequency distribution to extract the feature of radar waveform in automatic radar waveform recognition systems. However, limited research effort has been devoted to studying the bandwidth variations of the communication signals.

In this paper, the Gaussian pulse shaping signal is analyzed by Choi-Williams time frequency analysis which has good cross-term suppression characteristics [12]. It shows that the signal has a time-varying spectrum, i.e., the signal’s bandwidth is not constant, but varies with time in the passband. This brings an opportunity to suppress the co-channel interferences with time varying filter whose pass region is designed according to the desired signal’s energy distribution. For example, in a time frequency area Δ within the passband, the desired signal’s energy approximates to zero, but the energies of interferences are not negligible. In this case, if the received signal passes through a classical bandpass filter, the filter will collect all the energies in area Δ, which are mainly interferences. However, if the received signal passes through the time-varying filter, the filter will filter out area Δ and the strong interferences can be suppressed. Therefore, the time-varying filter can provide better SINR performance.

Various schemes for time-varying filters have been proposed, including the explicit time-varying filter [13], the implicit time-varying filter [13], the variable bandwidth filter [14], the time-frequency projection filter [15], and the adaptive filter [16,17]. In an adaptive filter, the received noisy signal is subjected to filtering and the filtered output is compared with a desired signal to compute the error. The task of the adaptive algorithm is to search for an optimal solution that minimizes the error. The adaptive algorithms include the gradient-descent-based search optimization algorithm, such as least mean square algorithm (LMS) [18], and the nongradient evolutionary algorithm, such as genetic algorithm (GA) [19] and particle swarm optimization algorithm(PSO) [20]. Quadri et al. [21,22] compared the performances of GA, PSO and LMS algorithms, and concluded that GA and PSO performed better than LMS for the case of AWGN corrupted signal, but for non-linear random noise PSO outperformed the other two algorithms.

Different filters have different advantages, disadvantages and applicable scenes. The short time Fourier transform (STFT)-based implicit time varying filter is easy to implement, but it has the problem of bad time frequency concentration and resolution [23,24,25]. The variable bandwidth filter-based on the extended Fourier transform (EFT) is suitable for the signal whose pass region is a band with clearly defined continuous upper bound and lower bound [26,27]. Hence if the pass region includes an area without clear bound, the variable bandwidth filter is not applicable. The time frequency projection filter could process any shape of time frequency pass region, but there exists an obstacle to find the orthogonal subspace to construct the signal with a given TF support [28]. As for Wiener and Kalman filter, the adaptive algorithm needs multiple iterations, which affects the processing time. Moreover, it is effective to suppress the white noise rather than co-channel interference [29]. Therefore, the explicit time varying filter is adopted in this paper, and the key task is to determine the pass region and the masking threshold.

The main contributions of this paper are summarized as follows.

- The time frequency distributions of various communication signals are first analyzed via Choi-Williams analysis. The quadrature modulated signals’ center frequency and bandwidth are not constant, but vary with time in the passband, and their distributions are irregular. Hence, it is a challenge to design the pass region for the time-varying filter. Fortunately, the distributions of the baseband and the binary modulated pulse shaping signals are regularly time-varying, which facilitates the pass region generation procedure.
- The equivalence of baseband and RF power spectrum is obvious with Fourier transform. However, since the time frequency distribution is preprocessed by the analysis window, the window length will affect the equivalence of the time frequency distribution of the baseband and RF signals. Two methods are proposed to guarantee the consistence of the time frequency distribution of the baseband and RF signals by adjusting the window length and zero padding.
- To suppress the non-orthogonal or co-channel interference, the explicit linear time varying filter is applied to mitigate the interference according to the energy distribution of the desired signal. Correspondingly, a masking threshold constrained time varying filter is developed with the optimal masking threshold determined by the minimum BER criterion.

The remainder of this paper is organized as follows. Section 2 describes the methodology of this paper, including the Choi-Williams analysis methods and the principle of the explicit time-varying filter; Section 3 exploits the time frequency distribution of various communication signals; Section 4 proposes the pass region generation method and the optimal masking threshold determination criterion; Section 5 applies the proposed masking threshold time-varying filter to suppress the co-channel interference; and Section 6 finally draws a conclusion.

## 2. Concept of Time Frequency Analysis and Processing

Time frequency analysis concerns the signals with time-varying frequency content. Such signals are best described by the time-frequency distribution on the time frequency plane. The time frequency distribution theories can be traced back to 1940s, when the researchers studied spectrogram with STFT [12]. With the development of time frequency theories, there are mainly two kinds of time frequency distributions. The first is the linear time frequency distribution which is developed from Fourier transform, such as short time Fourier transform [30] and Gabor transform [31]. The second is the bilinear time frequency distribution which is based on the representation of signals’ energy. The classical bilinear time frequency distribution is Wigner distribution, but it has the disadvantage of cross terms which affect the time frequency representation for the multiple-component signals. In 1966, Cohen [12] introduced the core function and expressed the time frequency distribution in a general form, whereas Choi and Williams argued that the cross terms could be minimized by choosing a proper core function [32]. Since Choi-Williams distribution is chosen as the time frequency analysis tool due to its advantage in suppressing spurious terms, this part will introduce Choi-Williams time frequency distribution in detail. As for STFT and Wigner-Ville distribution, please refer to [12] for more details.

### 2.1. Choi-Williams Distribution

Assume the signal to be analyzed is s(u), Cohen [12] introduced the core function ϕ(θ,τ), and defined the general form of time frequency distribution of signal s(u) as

(1) $$\begin{array}{cc} P(t,\omega )= & \frac{1}{4\pi^{2}}\;\int \int \int \;e^{-j\theta t-j\tau \omega +j\theta u}\phi \left(\theta ,\tau \right)s^{*}\left(u-\frac{\tau }{2}\right)s\left(u+\frac{\tau }{2}\right)dud\tau d\theta \end{array}$$

where P(t,ω) is the time frequency distribution of signal s(u), *t* and ω are the instant time and instant frequency respectively. θ and τ are two parameters of the core function ϕ. Based on the general form of time frequency distribution, Choi and Williams argued that instead of devising procedures to eliminate the cross term from the Wigner distribution, one could devise a distribution of which the spurious values were minimal. Consider a signal which is made up of components $s_{k}\left(t\right)$,

(2) $$s\left(t\right)=\sum_{k=1}^{N}s_{k}\left(t\right)$$

Substituting (2) into (1), one obtains that

(3) $$P\left(t,\omega \right)=\sum_{k=1}^{N}P_{kk}\left(t,\omega \right)+\sum_{k,l=1,k\neq l}^{N}P_{kl}\left(t,\omega \right)$$

where

(4) $$\begin{array}{cc} P_{kl}\left(t,\omega \right)= & \frac{1}{4\pi^{2}}\;\int \int \int \;e^{-j\theta t-j\tau \omega +j\theta u}\phi \left(\theta ,\tau \right)\cdot s_{k}^{*}\left(u-\frac{\tau }{2}\right)s_{l}\left(u+\frac{\tau }{2}\right)dud\tau d\theta \end{array}$$

Choi and Williams realized that by a judicious choice of the kernel, one could minimize the cross terms while retaining the desirable properties of the self terms. They found a particularly good choice for the kernel [32],

(5) $$\phi \left(\theta ,\tau \right)=e^{-\theta^{2}\tau^{2}/\sigma }$$

where σ is a constant. Substituting (5) into (1) and integrating over θ, one obtains

(6) $$\begin{array}{cc} P_{cw}\left(t,\omega \right)= & \frac{1}{4\pi^{3/2}}\;\int \int \;\frac{1}{\sqrt{\tau^{2}/\sigma }}e^{-[{\left(u-t\right)}^{2}/\left(4\tau^{2}/\sigma \right)]-j\tau \omega }s^{*}\left(u-\frac{\tau }{2}\right)s\left(u+\frac{\tau }{2}\right)dud\tau \end{array}$$

The ability to suppress the cross terms comes by means of controlling σ. From (6), each value of the time-indexed autocorrelation function $s^{*}\left(t-\tau /2\right)s\left(t+\tau /2\right)$ is obtained from a set of neighboring samples with selective weights $e^{-[{\left(u-t\right)}^{2}/\left(4\tau^{2}/\sigma \right)]-j\tau \omega }$, so that $s^{*}\left(u-\frac{\tau }{2}\right)s\left(u+\frac{\tau }{2}\right)$ has a large weight when *u* is close to *t*, and a small weight when *u* is far away from *t*. Furthermore, the size of the neighbor set is controlled by the variable τ, so that the size is increased for a large value of τ and decreased for a small value of τ, and σ can be used to control the relative importance of τ. By designing the weight, Choi and Williams reduced the spurious terms effectively.

**Figure 1:**
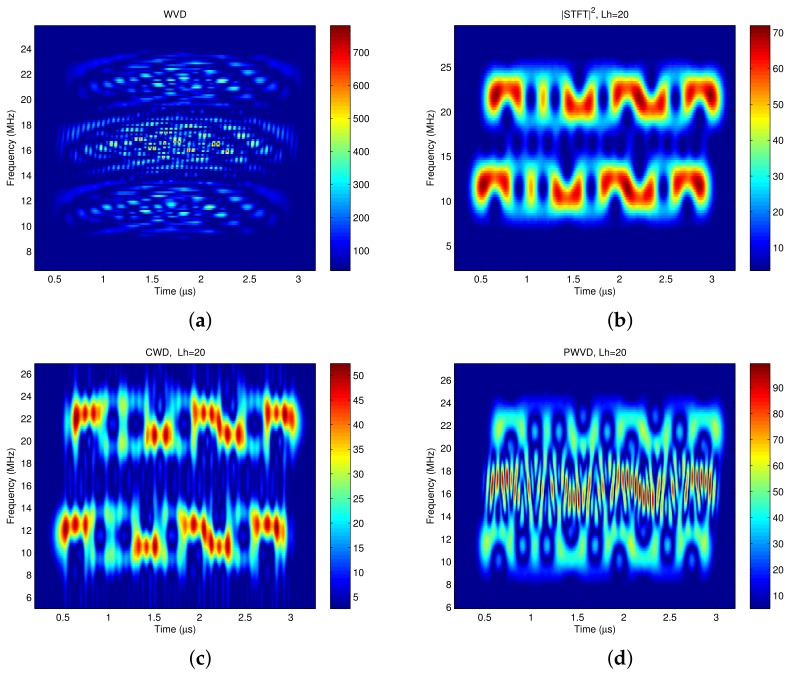
The time frequency distribution of a 4-QAM pulse shaping signal. (**a**) WVD; (**b**) STFT; (**c**) CWD; (**d**) SPWVD.

Figure 1 shows the time frequency distributions of a 4-QAM modulated signal which is the addition of two separated signals with center frequencies of 11.52 MHz and 21.52 MHz, respectively. The four time frequency analysis methods are WVD [12], STFT [12], CWD [32], and SPWVD (Smoothed Pseudo Wigner-Ville Distribution, [33]). The simulation parameters are listed in Table 1.

In principle, there should be only two signal components on the time frequency plane whose center frequencies are 11.52 MHz and 21.52 MHz with the bandwidths of 5 MHz, respectively. There should be no cross terms exist in other positions. Figure 1b shows that the linear time frequency method STFT does not produce cross terms. However, all of the other three bilinear time frequency methods produce cross terms, which are shown in Figure 1a,c,d, respectively. The cross-term problem is most severe in WVD , whereas CWD shows the best cross-term suppression ability. Although the STFT has no cross-terms, its time frequency concentration and resolution perform bad as a linear time frequency analysis method. Hence CWD is the best choice in the following time frequency analysis for the communication signals.

### 2.2. Explicit Time-Varying Filter Implementations

The time-varying filter could be applied by exploiting the energy concentration feature of the signal in two-dimensions instead of only one. In what follows, the principle of the explicit time varying filter is described. The explicit time-varying filter is a linear time varying (LTV) system, and the input-output relation of a discrete-time LTV system *H* is [13]

(7) $$z\left(n\right)=H\left[y\left(n\right)\right]=\sum_{n^{\prime }=-\infty }^{+\infty }h\left(n,n^{\prime }\right)y\left(n^{\prime }\right)$$

where $h(n,n^{\prime })$ is the impulse response of *H*, and y(n) and z(n) are the input and output of the time-varying filter, respectively.

For the underspread system, the linear time-varying filter could be expressed based on the general Weyl symbol (GWS), where the GWS of the LTV system *H* in continuous time is defined as

(8) $$\begin{array}{c} L_{H}^{\left(\beta \right)}\left(t,f\right)=\int_{-\infty }^{+\infty }h\left(t+\left(\frac{1}{2}-\beta \right)\tau ,t-\left(\frac{1}{2}+\beta \right)\tau \right)e^{-j2\pi f\tau }d\tau \end{array}$$

where *t* and τ are time variables, β is the real valued parameter controlling the time shifting of τ, and $L_{H}^{\left(\beta \right)}\left(t,f\right)$ is the GWS of system *H*. The special cases β=0 and β=1/2 give the Weyl symbol and Zadeh’s time-varying transfer function, respectively. The GWS with arbitrary β is not easily reformulated in a discrete-time setting. However, for β=1/2, the discrete-time formulation is given by

(9) $$L_{H}^{\left(1/2\right)}\left(n,\theta \right)=\sum_{m=-\infty }^{+\infty }h\left(n,n-m\right)e^{-j2\pi \theta m}$$

where *m* and *n* are discrete time variables, and θ denotes the normalized frequency. The discrete-time Zadeh filter is defined by setting $L_{H}^{\left(1/2\right)}\left(n,\theta \right)=M\left(n,\theta \right)$, where M(n,θ) is a time frequency weight function and can be obtained from the time frequency representation of the desired signal $P_{cw}\left(n,\theta \right)$. The impulse response of the Zadeh filter is obtained as

(10) $$h\left(n,n^{\prime }\right)=\int_{-1/2}^{1/2}M\left(n,\theta \right)e^{j2\pi \theta (n-n^{\prime })}d\theta$$

Therefore, the linear time-varying filter design problem can be simplified into constructing the indicator function M(n,θ) of the pass region *R*.

(11) $$M\left(n,\theta \right)=\left\{\begin{array}{cc} 1 & (n,\theta )\in R\\ 0 & (n,\theta )\notin R \end{array}\right.$$

where the pass region *R* is the time-frequency area in which the desired signal’s energy is not less than γ percent of the maximum value. It is defined according to the masking threshold γ, i.e., $R:\left\{n,\theta \right\}\in \{|P_{cw}\left(n,\theta \right)|\geq \gamma P_{cmax}\}$. $P_{cw}\left(n,\theta \right)$ is the desired signal’s time-frequency distribution, which is Choi-Williams distribution in this paper. $P_{cmax}$ is the maximum value of the desired signal’s time-frequency distribution, i.e., $P_{cmax}=\max\{|P_{cw}\left(n,\theta \right)\left|\right\}$.

## 3. Time-Frequency Distribution of Communication Signals

### 3.1. Time-Frequency Distribution of the Binary Modulated Signal

**Figure 2:**
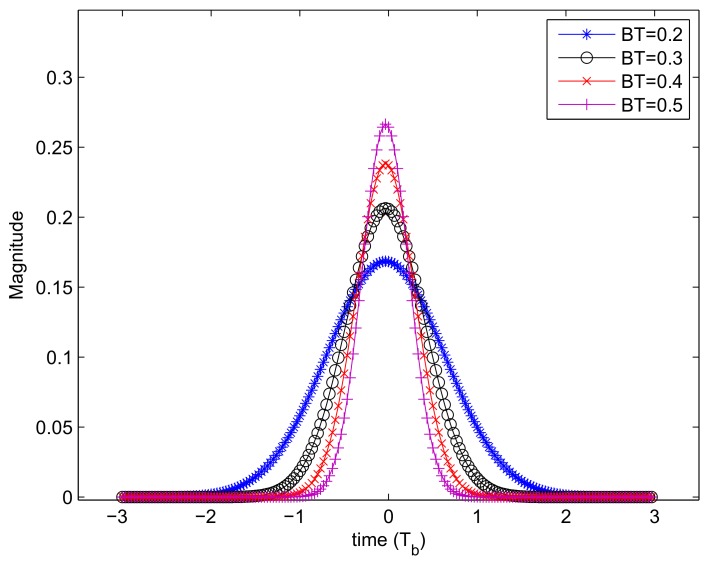
The Gaussian pulse waveform with different BTs.

**Figure 3:**
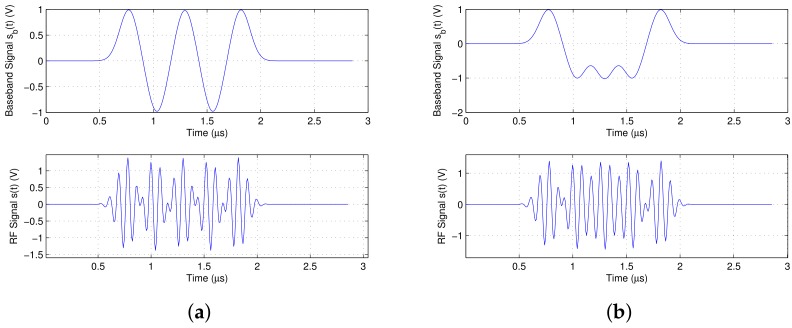
The baseband waveform and RF waveform of the pulse shaping signal. (**a**) Data stream {1,-1,1,-1,1}; (**b**) data stream {1,-1,-1,-1,1}.

In a binary modulated system, e.g., a BPSK modulation system, the baseband signal is denoted as $s_{b}\left(t\right)$. Then the modulated signal is expressed as

(12) $$s\left(t\right)=s_{b}\left(t\right)e^{j2\pi f_{0}t}$$

where $f_{0}$ is the center frequency of the modulated signal. In a communication system, the transmitted signal is pulse shaped at the baseband, and the pulse shaping filter is usually a raised cosine roll-off filter or a Gaussian filter. The waveform of the Gaussian pulse is plotted in Figure 2 with different BT values, where BT is the product of the Gaussian pulse’s 3 dB bandwidth and the symbol duration. It is mentioned that the Gaussian pulse has no zero-crossing points, and it does not satisfy the zero-ISI criterion. However, thanks to its best time frequency concentration, it is still widely applied in the pulse shaping system. Figure 3a illustrates the pulse shaped baseband signal when the transmitted data sequence is [1,-1,1,-1,1]. The pulse shaping filter is a Gaussian filter with BT = 0.4, and the center frequency of its corresponding modulated signal is 11.52 MHz. Similarly, Figure 3b depicts the pulse shaped baseband signal and its modulated signal for transmitted data sequence [1,-1,-1,-1,1].

It is shown that the magnitude of the baseband signal changes with time when the continuous two data are different, such as [1,-1] or [-1,1]. The absolute value of the magnitude reaches its maximum at each half symbol period, and falls to zero at the end of each symbol period. Therefore, the energy of the signal follows the same variation. In contrast, if the continuous two data are the same, such as [1,1], the magnitude of the baseband signal remains positive for the whole two symbol periods. However, its magnitude still reaches its maximum value at each half symbol period, and falls to a smaller value at the end of each symbol period. Apply Fourier transform to the above mentioned signal, and the nominal bandwidth is obtained, which is 3 dB bandwidth for the Gaussian pulse. In current communication systems, engineers design bandpass filters or low-pass filters according to the nominal bandwidth, and filter the signal at radio front end or baseband, respectively. By bandpass or low-pass filter, the desired signal in the pass-band is retained and the interference and noise outside the pass-band are suppressed. However, the interference and noise in the pass-band will pass through the filter.

**Figure 4:**
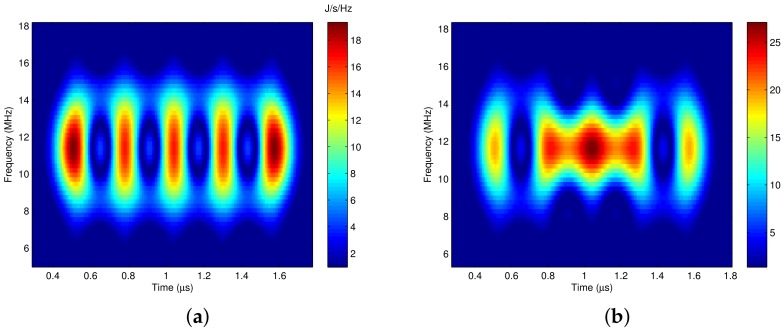
The time frequency distribution for pulse shaping signals. (**a**) Data stream {1,-1,1,-1,1}; (**b**) data stream {1,-1,-1,-1,1}.

The time frequency distribution of the Gaussian pulse shaping signal demonstrates that the bandwidth of the binary modulated pulse shaping signal is not constant, but varies with time, which is shown in Figure 4a,b. Figure 4a is the time frequency distribution corresponding to transmitted data [1,-1,1,-1,1]. It shows that if the continuous two data are different, the bandwidth of the signal reaches its maximum at the center of each symbol period, and shrinks to its minimal at the edge of each symbol period. Figure 4b is the time frequency distribution for transmitted data [1,-1,-1,-1,1], the bandwidth of the signal still reaches its maximum at each half symbol period, and shrinks narrower at the end of each symbol period. In conclusion, the binary modulated pulse shaping signal has a time-varying spectrum, and the variation shows certain regularity.

### 3.2. Time Frequency Distribution of Quadrature Modulated Signal

To improve the spectral efficiency, the modulation types used in current communication are quadrature modulation, such as QPSK and M-QAM. In a single carrier pulse shaping quadrature modulation system, the two pulse shaped signals for in-phase and quadrature paths are denoted as I(t) and Q(t), respectively. Define $s_{b}\left(t\right)=I\left(t\right)+jQ\left(t\right)$, then $s_{b}\left(t\right)$ is reformulated as $\sqrt{I^{2}\left(t\right)+Q^{2}\left(t\right)}e^{j\phi (t)}$, where ϕ(t) is the instant phase of signal $s_{b}\left(t\right)$, and ϕ(t)=atan{Q(t)/I(t)}. The modulated signal is expressed as

(13) $$s\left(t\right)=s_{b}\left(t\right)e^{j2\pi f_{0}t}=\sqrt{I^{2}\left(t\right)+Q^{2}\left(t\right)}e^{j(2\pi f_{0}t+\phi \left(t\right))}$$

where $f_{0}$ is the center frequency of signal s(t). From (13), ϕ(t) affects the center frequency of signal s(t), which makes it deviating relative to the carrier frequency $f_{0}$. The frequency deviation is denoted as ν(t), and then (13) is reformulated as (14),

(14) $$s\left(t\right)=\sqrt{I^{2}\left(t\right)+Q^{2}\left(t\right)}e^{j2\pi (f_{0}+\nu \left(t\right))t}$$

where ν(t) is determined by I(t) and Q(t), as shown in (15). $I^{\prime }\left(t\right)$ and $Q^{\prime }\left(t\right)$ represent the first order differentials of I(t) and Q(t), respectively.

(15) $$\nu \left(t\right)=\frac{1}{2\pi }\frac{Q^{\prime }\left(t\right)I\left(t\right)-I^{\prime }\left(t\right)Q\left(t\right)}{I^{2}\left(t\right)+Q^{2}\left(t\right)}$$

There are three cases:

**Case 1**. The magnitude of I(t) and Q(t) are constant in one symbol period. In this case, $I^{\prime }\left(t\right)=0$, and $Q^{\prime }\left(t\right)=0$. Then according to (15), ν(t)=0. The center frequency of the modulated signal does not deviate, it retains $f_{0}$ all the time.

**Case 2**. The magnitude of I(t) and Q(t) are not constant in one symbol period, but they satisfy the relationship I(t)=±Q(t). If I(t)=Q(t), replace Q(t) with I(t), and then the result $Q^{\prime }\left(t\right)I\left(t\right)-I^{\prime }\left(t\right)Q\left(t\right)=I^{\prime }\left(t\right)I\left(t\right)-I^{\prime }\left(t\right)I\left(t\right)=0$ is obtained, i.e., ν(t)=0. If I(t)=-Q(t), replace Q(t) with -I(t), and then $Q^{\prime }\left(t\right)I\left(t\right)-I^{\prime }\left(t\right)Q\left(t\right)=-I^{\prime }\left(t\right)I\left(t\right)-I^{\prime }\left(t\right)\left[-I\left(t\right)\right]=0$, i.e., ν(t)=0.

**Case 3**. The magnitude of I(t) and Q(t) are not constant, and I(t)≠±Q(t). In this case, $Q^{\prime }\left(t\right)I\left(t\right)-I^{\prime }\left(t\right)Q\left(t\right)\neq 0$, thus ν(t)≠0. The center frequency of the modulated signal varies with time.

**A The Time Frequency Distribution of the 4-QAM Modulated Signal**

**Figure 5:**
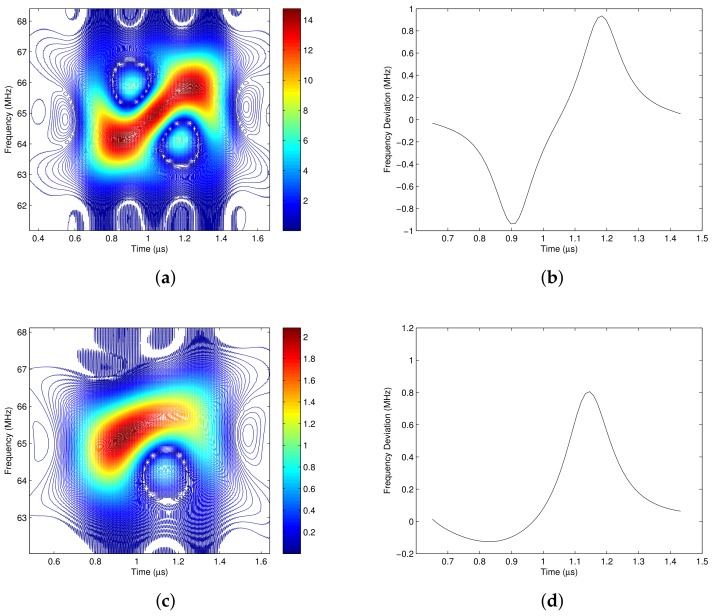
Time-frequency distribution and frequency deviation of 4-QAM modulated pulse shaping signals. (**a**) The time frequency distribution of {-1-i,-1+i,-1-i}; (**b**) The frequency deviation of {-1-i,-1+i,-1-i}; (**c**) The time frequency distribution of {1-i,1-i,1+i}; (**d**) The frequency deviation of {1-i,1-i,1+i}.

In a single carrier pulse shaping system, the frequency deviation ν(t) is determined by I(t) and Q(t), where I(t) and Q(t) are associated with *M* continuous transmitted data. Therefore I(t) and Q(t) have $2^{2M}$ kinds of combination, which makes the signal have $2^{2M}$ kinds of time-frequency energy distributions. It is considered that the pulse shaped waveform of each symbol is mainly affected by its two neighbor symbols, thus M=3. Therefore, there are 64 kinds of energy distributions for different I(t) and Q(t). However, by simulation, it is found that some of them are the same, and finally there are totally 16 kinds of energy distributions. Figure 5 illustrates two of them, where the continuous three symbols for Figure 5a are [-1-i,-1+i,-1-i], and the continuous three symbols for Figure 5c are [1-i,1-i,1+i].

In Figure 5a,c, the red contours represent the high energy area, and the dark blue contours represent the low energy area. It shows that the signal’s energy concentration varies with time. Figure 5b is the frequency deviation ν(t) of signal s(t) when the transmitted symbols are [-1-i,-1+i,-1-i]. It shows that the variation of ν(t) is in accordance with the energy center of the signal’s time frequency distribution, which is shown in Figure 5a. This verifies the physical analysis of (15). According to Figure 5a, the allocated bandwidth is not occupied by signal s(t) all the time. In the dark blue and blank areas, the desired signal’s energies are very low, but the interference and noise are not negligible. Hence if the signal is filtered according to its energy distribution, more interference and noise could be filtered out while maintaining most of the desired signal’s energy.

In summary, the pulse shaped quadrature modulated signal also has a time-varying spectrum, but it has no clear regularity. If apply the time-varying filter to the quadrature modulated signal directly, it would be a challenge to design the pass region of the time-varying filter. In [34], we discussed the performance improvement of the time-varying filter with perfect pass region, but it is not practical since the perfect pass region of the QAM modulated signal couldn’t be obtained at the receiver.

**B The Time Frequency Distribution of the SC-FDMA Signal**

Besides the single carrier pulse shaping 4-QAM signal, the time frequency distribution of the SC-FDMA signal is also discussed here. Let the sub-carrier spacing be $\omega_{0}=2\pi /N$. For any subcarrier *k* of the signal x(n), the sum of adjacent subcarrier signals is expressed as

(16) $$\begin{array}{cc} {\tilde{x}}_{k}\left(n\right)= & x_{k}\left(n\right)+x_{k+1}\left(n\right)\\ = & X\left(k\right)e^{jk\omega_{0}n}+X\left(k+1\right)e^{j\left(k+1\right)\omega_{0}n}\\ = & \left[X\left(k\right)+X\left(k+1\right)e^{j\omega_{0}n}\right]e^{jk\omega_{0}n} \end{array}$$

where X(k) is the data modulated on the $k_{th}$ subcarrier. Define the real and imaginary part of $X\left(k\right)+X\left(k+1\right)e^{j\omega_{0}n}$ as $A_{k}\left(n\right)$ and $B_{k}\left(n\right)$, respectively, where $A_{k}\left(n\right)=X\left(k\right)+X\left(k+1\right)\cos\left(\omega_{0}n\right)$, $B_{k}\left(n\right)=X\left(k+1\right)\sin\left(\omega_{0}n\right)$. Define $\phi \left(n\right)=\mathrm{atan}\{B_{k}\left(n\right)/A_{k}\left(n\right)\}$, and then ${\tilde{x}}_{k}\left(n\right)$ is reformulated as

(17) $${\tilde{x}}_{k}\left(n\right)=\sqrt{A_{k}^{2}\left(n\right)+B_{k}^{2}\left(n\right)}e^{\left(j\left(k\omega_{0}n+\phi \left(n\right)\right)\right)}$$

The time-varying phase ϕ(n) affects the center frequency of ${\tilde{x}}_{k}\left(n\right)$. Define ν(n) as the first order difference of ϕ(n), ${\tilde{x}}_{k}\left(n\right)$ is reformulated as

(18) $${\tilde{x}}_{k}\left(n\right)=\sqrt{A_{k}^{2}\left(n\right)+B_{k}^{2}\left(n\right)}e^{\left(j\left(\left(k\omega_{0}+\nu \left(n\right)\right)n\right)\right)}$$

since $\phi \left(n\right)=\mathrm{atan}\{B_{k}\left(n\right)/A_{k}\left(n\right)\}$, then ν(n) is reformulated as (19), where D[·] represents the first order difference.

(19) $$\nu \left(n\right)=D\left[\phi \left(n\right)\right]=\frac{1}{2\pi }\frac{D\left[B_{k}\left(n\right)\right]A_{k}\left(n\right)-D\left[A_{k}\left(n\right)\right]B_{k}\left(n\right)}{A_{k}^{2}\left(n\right)+B_{k}^{2}\left(n\right)}$$

Equation (18) shows that the center frequency of each subcarrier is not constant but varies with time, which is due to the summation of adjacent subcarrier signals. From (19), the frequency deviation of each subcarrier is determined by $A_{k}\left(n\right)$ and $B_{k}\left(n\right)$, where $A_{k}\left(n\right)$ and $B_{k}\left(n\right)$ are associated with the data carried by subcarrier *k* and k+1. Hence the frequency deviation is random and the sum of multiple sub-carriers will cause the bandwidth variations.

**Figure 6:**
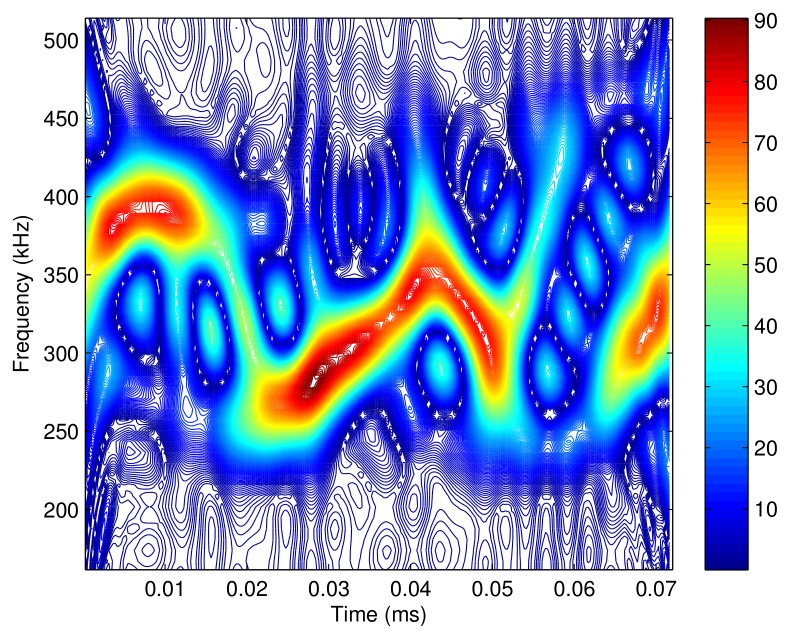
The time frequency distribution of the SC-FDMA signal.

Due to the randomness of the data modulated on each subcarrier, the spectrum of the SC-FDMA signal also has no regularity as shown in Figure 6. It would be a hard work to extract the effective pass region from the received signal, which has been contaminated by the noise. One solution is to determine the pass region by the energy distribution of the contaminated received signal. When the noise power is not strong enough to modify the desired pass region, we can use the time-frequency distribution of the received signal to represent the desired pass region. However, when the powers of interference and noise are high, they affect the desired pass region severely, and it is impossible to extract the effective pass region from the received signal. In summary, in an SC-FDMA system, although the SC-FDMA signal has a time-varying spectrum, it is possible to take the advantage of the time-varying filter only when the interference and noise are not too strong, which has been analyzed in our previous work [35].

### 3.3. The Time Frequency Distribution of I/Q Separated Signals

From the time frequency analysis of the single carrier pulse shaping quadrature modulated signal, it indicates that the energy of the signal does not occupy the whole passband all the time. Its energy concentrates in some time frequency areas, and leaves some low-energy areas, as shown in Figure 5a,c. This provides us a chance to suppress the interference and noise in the low-energy areas with the time-varying filter, which is designed according to the energy concentration of the pulse shaping quadrature modulated signal.

However, the physical analysis and time-frequency distribution of the QAM modulated signal show that its energy concentration is determined by the in-phase and quadrature transmitted data. Since the transmitted data are random generated, the energy distribution of the QAM signal varies with the pulse shaped baseband signals I(t) and Q(t), where I(t) and Q(t) are associated with *M* continuous data. Here, *M* is decided by the truncated length of the impulse response of the Gaussian filter. Therefore, I(t) and Q(t) have $2^{2M-2}$ kinds of combination, which makes the signal have $2^{2M-2}$ kinds of time-varying spectrums. This makes it difficult to generate the pass region for the time-varying filter. One solution is to generate the pass region from the received signal. However, since the received signal has been contaminated by the interference and noise, the energy distribution of the received signal is quite different from the desired signal, hence it is hard to generate the pass region from the received signal directly. Another solution is to match the time-frequency distribution of the received signal with $2^{2M-2}$ local pass region templates, and choose the most matching pass region template as the pass region of the time-varying filter. However, this solution also has its disadvantages. On one hand, the computational complexity is high; on the other hand, the time-frequency distribution of the received signal is distorted by the noise, thus wrong pass region templates may be chosen as the pass region of the time-varying filter. Therefore, the uncertainty of the time-frequency distribution of the quadrature modulated signal makes it hard to apply the time-varying filter.

At the receiver, the quadrature modulated signal is demodulated by the demodulator and the signal is separated into an in-phase signal and a quadrature signal. If there is no noise or interference, the in-phase signal $I_{r}\left(t\right)=I\left(t\right)$, and the quadrature signal $Q_{r}\left(t\right)=Q\left(t\right)$. The time frequency distribution of the separated signal is just the time frequency distribution of the baseband pulse shaped signal, as shown in Figure 4a,b.

It is shown that the time frequency distribution of the separated I/Q signal has the following characteristics: (1) the energy center of the separated signal is zero without shifting. (2) the time frequency distribution shows a certain regularity. If the continuous two data are different, the bandwidth of the signal reaches its maximum value at the center of each symbol period, and shrinks to its minimal value at the edge of each symbol period. If the continuous two or *N* data are the same, the bandwidth of the signal retains wide in the whole two or *N* symbol periods, but still reaches its maximum value at the center of each symbol period, and shrinks narrower at the edge of each symbol period. Therefore, if the signal is time-varying filtered with the proposed pass region, more interference and noise could be suppressed with less distortion to the desired signal. Meanwhile, since the filter processing is implemented at the baseband, the complexity is reduced.

### 3.4. The Equivalence of RF and Baseband Analysis

Is it equivalent to analyze the time frequency distribution of the RF signal and the separated baseband signal? This section will firstly discuss the equivalence between the power spectrum of a quadrature modulated signal and the power spectrum of its corresponding baseband complex signal. The baseband complex signal is denoted as $s_{b}\left(t\right)=I\left(t\right)+jQ\left(t\right)$, and the baseband spectrum is $S_{b}\left(f\right)=F\left\{s_{b}\left(t\right)\right\}$. Then modulate $s_{b}\left(t\right)$ to carrier with frequency $f_{c}$, and the modulated RF signal is expressed as

(20) $$s\left(t\right)=s_{b}\left(t\right)e^{j2\pi f_{c}t}$$

Apply Fourier transform to signal s(t), the magnitude spectrum of the RF signal is obtained as

(21) $$\begin{array}{cc} S(f)= & F\left\{s_{b}\left(t\right)e^{j2\pi f_{c}t}\right\}=\int_{-\infty }^{+\infty }s_{b}\left(t\right)e^{j2\pi f_{c}t}e^{-j2\pi ft}dt\\ = & \int_{-\infty }^{+\infty }s_{b}\left(t\right)e^{-j2\pi (f-f_{c})t}dt=S_{b}\left(f-f_{c}\right) \end{array}$$

The baseband power spectrum is $P_{b}\left(f\right)={\left|S_{b}\left(f\right)\right|}^{2}$, and the power spectrum of the RF signal is $P\left(f\right)={\left|S\left(f\right)\right|}^{2}={\left|S_{b}\left(f-f_{c}\right)\right|}^{2}=P_{b}\left(f-f_{c}\right)$. It is shown that power spectrum of the modulated signal can be obtained by moving the baseband power spectrum from center frequency 0 to $f_{c}$. If the modulated signal is not complex but real, its power spectrum will appear at frequency $\pm f_{c}$ simultaneously. Thanks to the power spectrum shifting characteristics between the baseband signal and its modulated signal, implementing a bandpass filter at radio front end is equivalent to implementing a low-pass filter at the baseband.

However, the two dimensional time-frequency analyses between the baseband signal and the modulated signal are not equivalent. In section II, it is mentioned that there are two kinds of methods to analyze the signal in joint time-frequency domain. The first is linear analysis, such as STFT and Gabor transform. in which the signal is truncated by the analysis window before analyzing. While the second is bilinear analysis, and the main analysis method is Wigner distribution. Since the Wigner distribution has severe cross-term problems, researchers provide some improved methods to suppress the cross terms, such as Pseudo smoothing Wigner distribution and Choi-Williams distribution. In both of the two distributions, the signal is also truncated by the analysis window before analyzing.

However, when applying Fourier transform to the truncated signal, the truncated length (or window length) affects the equivalence between the baseband signal and the modulated RF signal. For the modulated RF signal, if the window length is not integer multiples of the carrier cycle, the transition point will be introduced at the edge of extension period when implementing FFT calculation. The transition point will lead to carrier leakage, which will cause distortion to the time frequency distribution of the RF signal. In this case, the time frequency distribution of the RF signal disaccords with the time frequency distribution of the baseband complex signal.

**Figure 7:**
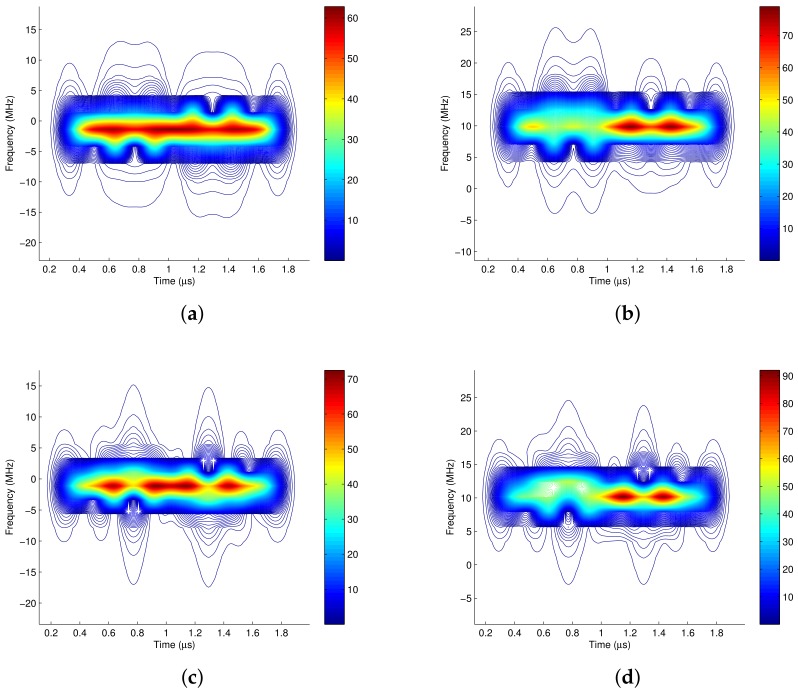
The effect of carrier leakage. (**a**) Baseband complex signal, $L_{h}$ = 40; (**b**) RF signal, $L_{h}$ = 40; (**c**) Baseband complex signal, $L_{h}$ = 50; (**d**) RF signal, $L_{h}$ = 50.

In Figure 7, the number of samples in each carrier cycle is $N_{sample}=30$. Figure 7a is the time frequency distribution of the baseband complex signal with the window length $L_{h}=40$, and Figure 7b is the time frequency distribution of the RF signal when the window length $L_{h}=40$. It is shown that the energy center of Figure 7b has changed compared to Figure 7a. Since the truncated length is not integer multiples of the carrier cycle, it is 1.1 times in this example, the time frequency distribution of the RF signal has been distorted. Figure 7c is the time frequency distribution of the baseband complex signal with the window length $L_{h}=50$, and Figure 7d is the time frequency distribution of the RF signal when the window length $L_{h}=50$. It also shows the non-equivalence between the time frequency distribution of the baseband signal and that of the RF signal when the window length is not integer multiples of the carrier cycle.

**Figure 8:**
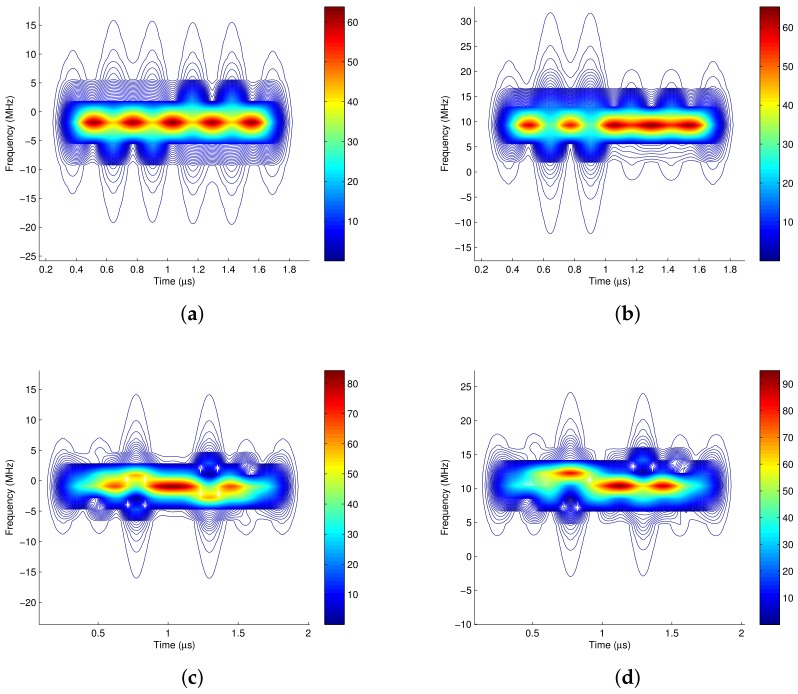
Realizing time-frequency equivalent analysis of baseband and RF by controlling the window length. (**a**) Baseband complex signal, $L_{h}$ = 30; (**b**) RF signal, $L_{h}$ = 30; (**c**) Baseband complex signal, $L_{h}$ = 60; (**d**) RF signal, $L_{h}$ = 60.

**Figure 9:**
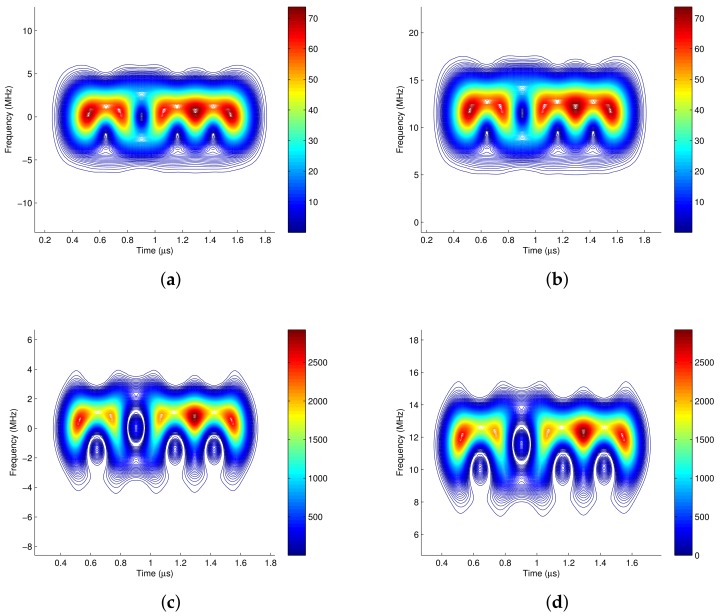
Realizing time-frequency equivalent analysis of baseband and RF by adding Hamming window and zero padding. (**a**) baseband complex signal, with hamming analysis window and zero padding, STFT, $L_{h}$ = 40; (**b**) RF signal, with hamming analysis window and zero padding, STFT, $L_{h}$ = 40; (**c**) baseband complex signal, with hamming analysis window and zero padding, CW, $L_{h}$ = 40; (**d**) RF signal, with hamming analysis window and zero padding, CW, $L_{h}$ = 40.

There are two methods to solve the non-equivalence problem between the time frequency distribution of the baseband signal and the RF signal:

(1) Let the window length be integer multiples of the carrier cycle, so that there is no carrier leakage problem. In this case, the time frequency analysis of the baseband signal and that of the RF signal are equivalent. For example, Figure 8a is the time frequency distribution of the baseband complex signal when the window length equals to the carrier cycle, i.e., $L_{h}=30$, and Figure 8b is the time frequency distribution of its corresponding RF signal when the window length is $L_{h}=30$. It is obvious that the time frequency distributions of them are equivalent. In Figure 8c,d, the window length is two multiples of the carrier cycle, i.e., $L_{h}=60$, It also shows the equivalence between the time frequency distribution of the baseband signal and that of the RF signal when the window length is integer multiples of the carrier cycle.

(2) Pad zeros before and after the truncated signal before Fourier transforming. The immediate benefit is the improvement of the analysis resolution in frequency domain. Fortunately, by zero padding, the equivalence between the time frequency distribution of the baseband complex signal and that of the RF signal is maintained even if the analysis window length is not integer multiples of the carrier cycle. Figure 9a is the STFT of the baseband complex signal when the truncated length is $L_{h}=40$, and Figure 9b is its corresponding STFT of the RF signal. It validates the equivalence of the time frequency distribution of the baseband signal and the RF signal, even if the truncated length is not integer multiple of the carrier cycle. Figure 9c,d are the Choi-Williams distribution of the baseband and RF signal, respectively, which also validates the equivalence with zero padding, even if the truncated length is not integer multiples of the carrier cycle.

In what follows, the Zero padding method is adopted to generate the signal’s Choi-Williams time frequency distribution.

## 4. Masking Threshold Constrained Time-Varying Filter

### 4.1. The Pass Region Generation

For the single carrier pulse shaping signal, the time frequency distribution of the I/Q separated baseband signal shows a regularity. Based on this regularity, the fixed pass region is generated to design the time-varying filter, where the pass region generation procedure is:

- Step 1: Generate the 1/−1 alternate data sequence. If the processing window length of the time-varying filter is $NT_{b}$, the number of the generated data is *N*.
- Step 2: The data sequence passes through the pulse shaping filter, and the pulse shaped baseband signal $s_{o}\left(n\right)$ is generated.
- Step 3: Calculate the time frequency distribution of $s_{o}\left(n\right)$, and obtain $S_{o}\left(n,\upsilon \right)$.
- Step 4: Generate the pass region *R* with the pre-defined masking threshold γ. $R:\left\{n,\upsilon \right\}\in \left\{\left|S_{o}\left(n,\upsilon \right)\right|\geq \gamma S_{o}^{max}\right\}$, where $S_{o}^{max}$ is the maximum value of $S_{o}\left(n,\upsilon \right)$, i.e., $S_{o}^{max}=max\left\{\left|S_{o}\left(n,\upsilon \right)\right|\right\}$.
- Step 5: Construct the time frequency weighting function M(n,υ) according to the pass region *R*, which is shown in (11).

**Figure 10:**
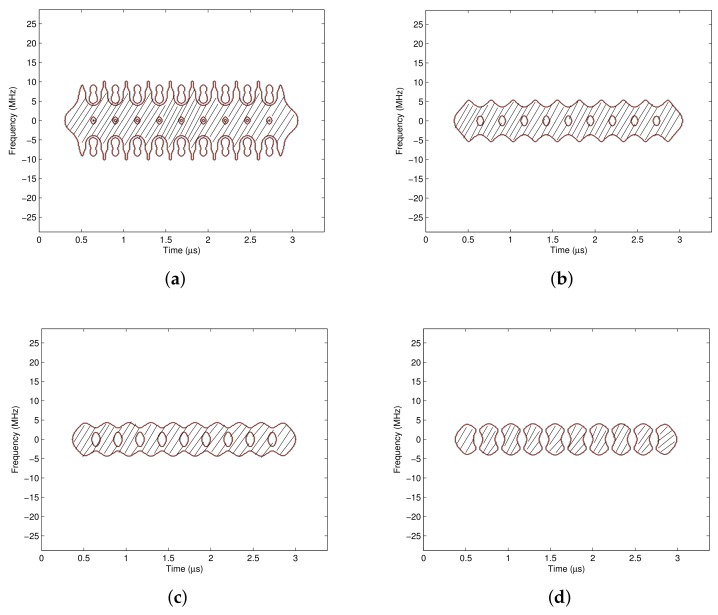
The pass regions with different masking thresholds. (**a**) The pass region with γ = 0.04; (**b**) The pass region with γ = 0.1; (**c**) The pass region with γ = 0.2; (**d**) The pass region with γ = 0.26.

Figure 10 shows the pass region with different masking thresholds γ. It illustrates that a smaller masking threshold allows a wider pass region, and more energies of the desired signal are collected. However, unfortunately, the interference and noise in the pass region will pass through the time-varying filter. As the masking threshold γ grows, the pass region shrinks, and the energies at the edge of each symbol period are filtered out gradually. If the energy center of the interference signal is just located at the edge of each symbol period, more interference could be suppressed, but the desired signal will be distorted severely. Therefore, there should be an optimal masking threshold γ with less desired signal distortion and more interference suppression.

The optimal masking threshold $\gamma_{opt}$ is determined by the minimum bit error rate criterion.

(22) $$\gamma_{opt}=\arg\min_{\gamma }\left\{\mathrm{BER}\right\}$$

**Figure 11:**
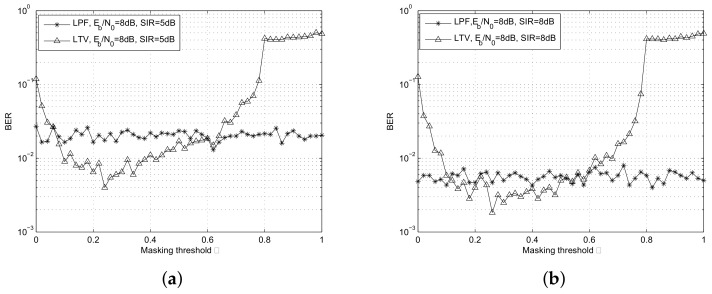
BER varies with different masking thresholds. (**a**) $E_{b}/N_{0}$ = 8 dB, SIR = 5 dB; (**b**) $E_{b}/N_{0}$ = 8 dB, SIR = 8 dB.

Figure 11 shows how the BER of the desired signal varies with the masking threshold γ. In the figure, LPF represents the low pass Butterworth filter with a bandwidth of 2.5 MHz, and the filter order is 4; LTV represents the GWS based linear time varying filter. In the simulation, the $E_{b}/N_{0}$ is set to 8 dB, and the power ratios of the desired signal to the interference signal are 5 dB and 8 dB, respectively. The analysis window is the Hamming window with length $T_{b}$. It is shown that the BER with the LTV filter is worse than that with the LPF filter when the masking threshold is too small or too big. Because when the threshold is too small, the pass region contains more time frequency areas on the time frequency plane. In this case, although most of the energies of the desired signal are collected, the interference suppression effect is not obvious. In contrast, when the threshold is too big, the pass region shrinks, the interference locating at the edge of each symbol period can be suppressed. However, the distortion of the desired signal overwhelms the interference suppression, hence the BER with the LTV filter is worse than that with the LPF filter. From Figure 11a, the optimal masking threshold $\gamma_{opt}$ = 0.24 when SIR is 5 dB, and $\gamma_{opt}$ = 0.26 when SIR is 8 dB.

### 4.2. Pass Region with Different Time Frequency Analysis Windows

**Figure 12:**
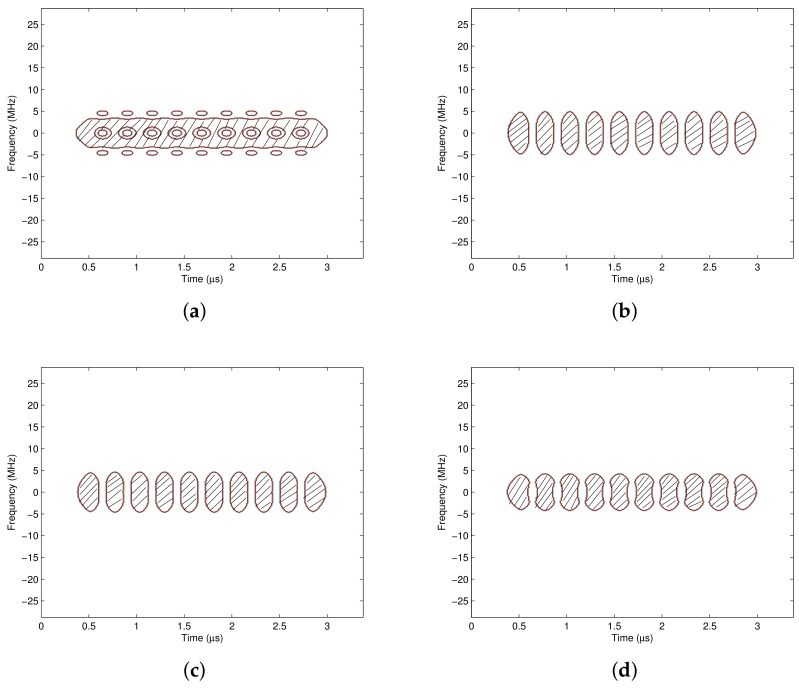
The pass regions with different analysis windows. (**a**) The pass region with the rectangular window; (**b**) The pass region with the Gaussian window; (**c**) The pass region with the Blackman window; (**d**) The pass region with the Hanning window.

The pass region is generated according to the signal’s time frequency distribution, of which time frequency concentration and resolution are affected by the analysis window. In this section, the influences of the window type and the window length on the pass region are discussed. Figure 12 shows the pass region obtained with different analysis windows, where the time frequency distribution method is CWD and the masking threshold γ is 0.26. It indicates that the time frequency concentration and resolution with the rectangular window are both bad, where the energies of the signal are dispersive on the time frequency plane, and the pass region contains more time frequency areas. If the time-varying filter is designed according to the pass region in Figure 12a, more interferences will be collected, which makes the interference suppression performance bad. In contrast, the concentration and resolution of the signal’s time frequency distributions with the Gaussian window, the Blackman window and the Hanning window are similar, and the generated pass regions are also similar. When γ=0.26, the high energy areas of the interferences are excluded from the pass region, and the interference suppression ability is stronger than the pass region generated with the rectangular window.

**Figure 13:**
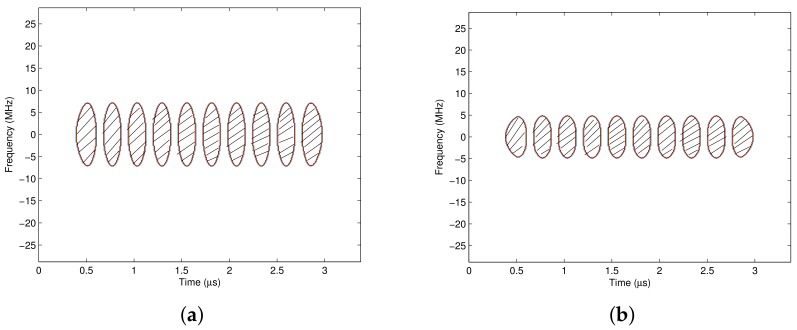

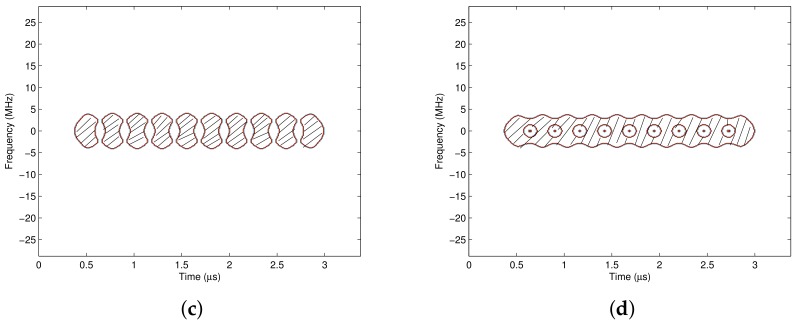
The pass regions with different window lengths. (**a**) The pass region with *L_h_* = 10; (**b**) The pass region with *L_h_* = 20; (**c**) The pass region with *L_h_* = 30; (**d**) The pass region with *L_h_* = 40.

Besides the window type, the analysis window length also affects the scope of the pass region. Figure 13 shows the pass regions with different window lengths, where the analysis window is the Hamming window. It indicates that when the window length is short, the time resolution is high but the frequency resolution is low. Accordingly, the low energy characteristic at the edge of each symbol period is obvious, but the bandwidth at the center of each symbol period is spreaded. As the window length grows, the time resolution is reduced whereas the frequency resolution is increased, and the low energy characteristic at the edge of each symbol period is not so obvious, but the bandwidth of each symbol becomes narrower. Hence the designer should choose an appropriate window length according to the energy distributions of the desired signal and the interference.

## 5. Time Frequency Distribution Based Co-Channel Interference Suppression

The time frequency distribution of the pulse shaping signal has low energy characteristics at the edge of each symbol period. Based on this feature, if the energy center of the co-channel interference is located at the edge of the desired signal’s symbol period, then the interference could be suppressed by the time varying filter of which the pass region is generated according to the energy distribution of the desired signal.

**Figure 14:**
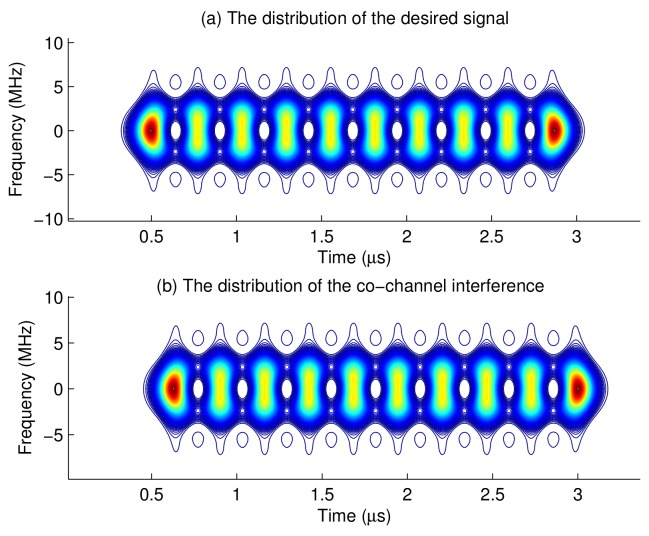
Time frequency distributions of the desired and co-channel interference signals.

Assume that the desired signal is $s_{i}^{c}\left(t\right)$, and the co-channel interference signal is denoted as $s_{i}^{d}\left(t\right)=s_{i}^{c}\left(t-T_{b}/2\right)$, where $T_{b}$ is the symbol period. The interference has half symbol period time difference from the desired signal. Figure 14 shows the time frequency distributions of the desired signal and the interference. It indicates that the high energy of the interference just locates at the low energy area of the desired signal, and vice versa. If apply the time varying filter according to the energy distribution of the desired signal, more co-channel interference can be suppressed.

The simulation parameters are listed in Table 2.

**Figure 15:**
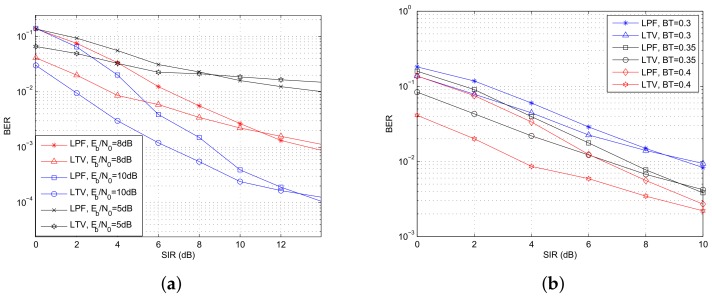
The BER performance with the proposed co-channel interference suppression method. (**a**) BER varies with different SIRs; (**b**) BER varies with SIRs with different BTs.

Figure 15a shows how the BER performance varies with SIRs with the LPF and the LTV filters, respectively. It indicates that the LTV filter provides better BER performance when the interference power is strong. Since the proposed time varying filter aims at mitigating the high energy area of the interference, the suppression ability is more obvious with stronger interference. Take $E_{b}/N_{0}$ = 10 dB for example, it shows that the LTV filter achieves better BER performance than the LPF filter, with an SIR gain of about 3 dB for strong co-channel interference (SIR < 4 dB), and 2.5 dB for moderate co-channel interference (SIR > 4 dB). However, when the interference is weak, the BER with the LTV filter is high, which is because that the distortion of the desired signal overwhelms the suppression to the interference in this case. Figure 15a plots three groups of curves that BER varies with SIRs when $E_{b}/N_{0}$s are set to be 5 dB, 8 dB and 10 dB, respectively. It shows that the performance crossing point varies with different $E_{b}/N_{0}$s. The three performance crossing points are 8.5 dB, 11 dB and 13 dB, respectively. The complexity of LTV filter is O($N^{3}$), and the complexity of LPF filter is O(*N*), which is shown in Table 3.

Figure 15b shows how the BT value affects the filter effect. It indicates that the performance crossing points are 8.5 dB and 9 dB, when the BTs are 0.3 and 0.35, respectively. When the BER is $2\times 10^{-2}$, the SIR gain for the three groups of curves are 0.5 dB, 1.5 dB and 3 dB, respectively. As shown in Figure 2, the low energy characteristic of the desired signal at the edge of each symbol period is more obvious with bigger BT, hence more advantages could be obtained with the LTV filter.

## 6. Conclusions

In this paper, the time frequency distributions of various communication signals are first analyzed via Choi-Williams analysis. The distributions of the baseband and binary modulated pulse shaping signals are time-varying and regular, which facilitates the pass region generation procedure. Then, the equivalence of the time frequency distributions of the baseband and RF signals is discussed, and two methods are proposed to guarantee the consistence by adjusting the window length and zero padding. In addition, the masking threshold constrained time-varying filter is applied to suppress the co-channel interferences, of which the pass region is generated according to the energy distribution of the desired signal. According to the simulation evaluations, the proposed time-varying filter can suppress the co-channel interference efficiently, especially when the interference power is strong.

## Figures and Tables

**Table 1:** Simulation Parameters.

Parameters	Values
Pulse shaping filter	raised cosine roll-off filter
Raised cosine roll-off factor	0.22
Modulation type	4-QAM
Symbol time period $T_{b}$	0.26042 μs
Truncated length of Raised cosine pulse	$6T_{b}$
Center frequency of the first signal	11.52 MHz
Center frequency of the second signal	21.52 MHz
Analysis window type	Hamming
Analysis window length	$4/3T_{b}$
σ for Choi-Williams distribution	1

**Table 2:** Simulation Parameters.

Parameters	Values
Gaussian pulse parameter BT	0.4
Truncated length of the Gaussian pulse	$6T_{b}$
Center frequency of the expected signal	800 MHz
Center frequency of the interference signals	800 MHz
Symbol time period $T_{b}$	0.26042 μs

**Table 3:** The Comparison between LTV filter and LPF filter.

Parameters	LTV Filter	LPF Filter
BER performance when $E_{b}/N_{0}$ = 10 dB, SIR = 4 dB	$3\times 10^{-3}$	$2\times 10^{-2}$
Complexity	O($N^{3}$)	O(*N*)

## Abbreviations

4-QAM	4th order Quadrature Amplitude Modulation
BER	Bit Error Rate
BPSK	Binary Phase Shift Keying
BT	Bandwidth Time product
CWD	Choi-Williams Distribution
D2D	Device-to-Device communications
$E_{b}/N_{0}$	Bit energy to noise spectral density ratio
GNSS	Global Navigation Satellite System
GWS	General Weyl Synbol
LPF	Low Pass Filter
LTV	Linear Time Varying
M-QAM	Mth order Quadrature Amplitude Modulation
QPSK	Quadrature Phase Shift Keying
SC-FDMA	Single Carrier-Frequency Division Multiple Access
SIR	Signal to Interference Ratio
SINR	Signal to Interference and Noise Ratio
SNR	Signal to Noise Ratio
STFT	Short Time Fourier Transform
WVD	Wigner-Ville Distribution
